# Fucoxanthin Exerts Cytoprotective Effects against Hydrogen Peroxide-induced Oxidative Damage in L02 Cells

**DOI:** 10.1155/2018/1085073

**Published:** 2018-11-15

**Authors:** Xia Wang, Yan-jun Cui, Jia Qi, Min-min Zhu, Tian-liang Zhang, Min Cheng, Shun-mei Liu, Guang-ce Wang

**Affiliations:** ^1^School of Public Health and Management, Weifang Medical University, Weifang 261053, China; ^2^Collaborative Innovation Center of Prediction and Governance of Major Social Risks in Shandong, Weifang Medical University, Weifang 261053, China; ^3^School of Bioscience and Technology, Weifang Medical University, Weifang 261053, China; ^4^Experimental Center for Medical Research, Weifang Medical University, Weifang 261053, China; ^5^School of Clinical Medicine, Weifang Medical University, Weifang, 261053, China; ^6^Institute of Oceanology, Chinese Academy of Sciences, Qingdao 266071, China

## Abstract

Several previous studies have demonstrated the excellent antioxidant activity of fucoxanthin against oxidative stress which is closely related to the pathogenesis of liver diseases. The present work was to investigate whether fucoxanthin could protect human hepatic L02 cells against hydrogen peroxide- (H_2_O_2_-) induced oxidative damage. Its effects on H_2_O_2_-induced cell viability, lactate dehydrogenase (LDH) leakage, intracellular reduced glutathione, and reactive oxygen species (ROS) contents, along with mRNA and protein relative levels of the cytoprotective genes including Nrf2, HO-1, and NQO1, were investigated. The results showed that fucoxanthin could upregulate the mRNA and protein levels of the cytoprotective genes and promote the nuclear translocation of Nrf2, which could be inhibited by the PI3K inhibitor of LY294002. Pretreatment of fucoxanthin resulted in decreased LDH leakage and intracellular ROS content but enhanced intracellular reduced glutathione. Interestingly, pretreatment using fucoxanthin protected against the oxidative damage in a nonconcentration-dependent manner, with fucoxanthin of 5 *μ*M demonstrating the optimal effects. The results suggest that fucoxanthin exerts cytoprotective effects against H_2_O_2_-induced oxidative damage in L02 cells, which may be through the PI3K-dependent activation of Nrf2 signaling.

## 1. Introduction

Reactive oxygen species (ROS), including free radical such as hydroxyl radical and non-free radical such as hydrogen peroxide (H_2_O_2_), are highly reactive byproducts derived from normal cell metabolism, especially from that of mitochondria [1]. Generally, appropriate generation of ROS might be essential for many cellular functions such as phagocytes killing and bacterial ingestion but detrimental to living organisms in the case of overproduction [2]. Oxidative stress represents an imbalance between pro-oxidants and antioxidants. The antioxidative status was found inversely correlated with the occurrence of numerous human diseases [3, 4]. Hepatic cells are rich in mitochondria and prone to generate ROS [5]. Role of oxidative damage in the pathogenesis of various liver injuries has been confirmed. It has been suggested that the overproduction of H_2_O_2_ contributes to the pathogenesis of many liver diseases such as hepatitis C virus infection, cholestasis and Wilson's disease etc [6]. Enzymatic antioxidants (such as glutathione (GSH) or glutathione-related enzyme system) and non-enzymatic ones are the two major systems to control ROS generation and counteract the oxidative damage [7]. Therefore, it has become an interesting and urgent topic for researchers to find excellent antioxidants for the prevention of liver diseases.

Many synthetic antioxidants, such as butylated hydroxytoluene (BHT) and butylated hydroxyanisole (BHA), have been used to slow down the processes of oxidation and peroxidation in food and pharmaceutical industries. However, their utilizations have been severely restricted due to their potential toxicity to human health because of their carcinogenic effects [8]. And BHT, when applied together with PG, resulted in joint pathology and liver enlargement [9]. Moreover, continuous use of some synthetic phenolic antioxidants exerted teratogenic and carcinogenic effects in laboratory animals and primates [10]. Therefore, natural antioxidants, with minimal side or toxic effects, have been drawing increasing attention from researchers in the past decades in viewing of consumers' preferences and concerns about health.

Carotenoids, natural pigments found in plants, algae, animals, etc., possess the antioxidant ability to protect cells and tissues from detrimental effects caused by ROS [11]. The role of carotenoids to protect against diseases caused by oxidants has been confirmed. Fucoxanthin, mainly existing in brown seaweed, accounts for more than 10% of total carotenoids production in nature [12]. It was speculated that its unique allenic bond and 5, 6-monoepoxide might be crucial for radical scavenging and protecting against cell damages induced by exposure to H_2_O_2_ or UV-B, with its high antioxidant activity probably attributed to the allenic bond [13].

Nuclear factor-erythroid 2-related factor 2 (Nrf2 or Nfe2l2) is a transcription factor sensitive to cellular stresses such as oxidative stress. Under normal physiological conditions, Nrf2 is mainly in cytoplasm and transcriptionally inactive due to proteasomal degradation mediated by Kelch-like ECH-associated protein I (Keap1) which acts as an inhibitor. Under oxidative stress, the sulfhydryl groups of Keap1 can be easily oxidized and its binding affinity to Nrf2 decreases, resulting in the translocation of Nrf2 from the cytoplasm to the nucleus and subsequent binding to antioxidant response elements (ARE), which could enhance the expressions of many stress-induced genes such as HO-1 and NQO1 [14]. It has been reported previously that several dietary compounds could protect cells from oxidative damage through Nrf2-ARE pathway [15].

Several studies have reported the antioxidant effect of fucoxanthin, mainly focusing on the scavenging activity against different free radicals (such as DPPH, ABTS, hydroxyl radical, hydrogen peroxide, and superoxide anion) and the ability to quench singlet oxygen [16–19] using cell-free system or its inhibitory effects on ROS production, DNA damage and cell apoptosis induced by H_2_O_2_, UV, and other factors using cell models [20–23]. H_2_O_2_, widely used to induce oxidative stress in in vitro models, could easily cross the cellular membranes and generate highly toxic hydroxyl radicals which could react with macromolecules such as DNA and proteins, thus leading to cellular damages [24]. As an essential organ responsible for detoxification and biochemical metabolisms, liver is susceptible to oxidative damage. Moreover, oxidative stress in normal rat hepatic cells in vitro induced by H_2_O_2_ was similar to that occurred in intact liver [25]. The human L02 cells, commonly accepted in vitro experimental model and used for exploring the pathogenesis of liver diseases [26], were also employed in the present work. Liu et al. suggested the participation of Nrf2-ARE pathway regarding the antioxidant activity of fucoxanthin in murine hepatic BNL CL.2 cells [27]. In consideration of the high incidence of liver diseases in China, we select fucoxanthin due to its high antioxidant activity to investigate its cytoprotective effects against H_2_O_2_-induced oxidative damage in hepatic L02 cells and whether it works through the Nrf2-ARE pathway.

## 2. Materials and Methods

### 2.1. Cell Culture and Treatments

Normal human hepatic cell line of L02, purchased from the Procell Life Science & Technology Co., Ltd. (Wuhan, Hubei Province, China), was cultured in RPMI 1640 medium (Solarbio, Beijing, China) containing 10% (v/v) fetal bovine serum (FBS), penicillin of 100 U/mL, and streptomycin of 100 *μ*g/mL in a humidified incubator, with CO_2_ as 5% and temperature as 37°C. Cells were transferred to appropriated multiwall plates and used for subsequent experiments when the confluence reached about 70%. Fucoxanthin (Sigma-Aldrich, Steinheim, Germany) was dissolved in DMSO and diluted with culture medium to various concentrations for following tests. Cells from experimental groups were incubated with fucoxanthin for 2 h prior to the oxidative stress induced by H_2_O_2_.

### 2.2. Cell Viability

Cell viability of L02 was evaluated through MTT assay. Briefly, cells were seeded into 96-well plates, with cell concentration as 1 × 10^5^ cells/well, and cultured in serum-free medium for 24 h. Then the cells from experimental groups were pretreated with fucoxanthin at different concentrations (final concentration as 1, 5, 10, or 20 *μ*M, respectively) or vitamin E (final concentration as 50 *μ*M) and cultured for 2 h, followed by exposure to H_2_O_2_ (final concentration as 200 *μ*M). After incubation for 24 h, MTT (final concentration as 0.5 mg/mL) was added into each well and incubation for another 4 h at 37°C was performed. Subsequently, the plate was centrifuged at a speed of 800 g for 5 min and the supernatant was discarded. Then the formazan crystals formed in each well were dissolved using 100 *μ*L DMSO and the absorbance was measured using a microplate reader (PowerWave XS, Bio-Tek, Winooski, VT, USA) at a wavelength of 540 nm. The relative cell viability was calculated by comparison with the absorbance of untreated control group.

### 2.3. Intracellular ROS Measurement

The intracellular ROS level was evaluated by the 2′,7′-dichlorodihy-drofluorescein diacetate (DCFH-DA) method. Cells (1 × 10^5^ cells/mL) were seeded into 96-well plates and cultured for 24 h. Then cells from experimental groups were subjected to various aforementioned concentrations of fucoxanthin or vitamin E (final concentration as 50 *μ*M) and cultured for 24 h, followed by treatment of 200 *μ*M H_2_O_2_ (final concentration) for 30 min at 37°C. After being washed with PBS, cells were treated with 10 *μ*M DCFH-DA for 20 min at 37°C in a humidified incubator. After the redundant DCFH-DA was removed, cells were washed carefully using PBS three times and the fluorescence intensities of DCF were determined using a fluorescence microplate reader (VICTRO3, PerkinElmer, Waltham, MA, USA), with excitation wavelength at 485 nm and emission wavelength at 535 nm. And the representative images concerning the levels of intracellular ROS were also captured using an inverted fluorescence microscope (DMI400B; Leica, Wetzlar, Germany) and the fluorescence intensities which reflects the contents of ROS were analyzed using IPP software (Version 6.0, Media Cybernetics, USA) and normalized to that of the control group.

### 2.4. Lactate Dehydrogenase (LDH) Leakage Assay

The LDH leaked in the culture was evaluated using a LDH activity assay kit (Beyotime Institute of Biotechnology, Shanghai, China) according to the supplier's instructions. Briefly, cells (1 × 10^5^ cells/mL) were seeded into 96-well plates and cultured for 24 h. Then the cells from experimental groups were treated with various aforementioned concentrations of fucoxanthin or vitamin E (final concentration as 50 *μ*M) and cultured for 2 h, followed by treatment of 200 *μ*M H_2_O_2_ (final concentration) for another 24 h at 37°C in a humidified incubator. The culture was subsequently mixed with the relevant kit reaction solution and incubated at 25°C. Finally, the LDH activity in culture was determined based on the absorbance at 450 nm using a microplate reader (PowerWave XS, Bio-Tek, Winooski, VT, USA). The LDH leakage rate was calculated by comparing with the maximum LDH activity control group.

### 2.5. Detection of Intracellular Reduced GSH

The experimental grouping and treatment were conducted as described in method 2.4. After incubation, cells were made into cell suspensions by trypsin digestion and washed with PBS three times, followed by homogenate using an ultrasonic cell disruption system (JY92-IIN, Xinzhi, Ningbo, China). Then the intracellular glutathione was measured using a glutathione determination kit (A006-2, Jiancheng, Nanjing, China) in accordance with the supplier's instructions. The intracellular glutathione was evaluated based on the absorbance at 420 nm using a fluorescence microplate reader (SpectraMax M5, MD, CA, USA), with excitation wavelength at 350 nm.

### 2.6. Real-Time Quantitative PCR

One additional group named L was added for real-time PCR and the following western blot along with the immunofluorescent assay. As to group L, cells were firstly incubated with fucoxanthin of 5 *μ*M for 1 h, followed by addition of LY294002 (a specific inhibitor of PI3K, final concentration as 20 *μ*M) and H_2_O_2_ of 200 *μ*M (final concentration). After being treated as aforementioned in method 2.4 and washed three times using PBS, cells derived from each group were collected and the total RNA was extracted using Trizol reagent (Invitrogen, Carlsbad, CA, USA), followed by the synthesis of cDNA by a commercial first strand cDNA synthesis kit (Takara, Otsu, Japan). The relevant cDNA was kept at −70°C before use. The primers, designed using software of Primer5, were synthesized by Takara. Then the triplicate cDNA samples, included in a reaction system of totally 20 *μ*L mainly containing approximately 50 ng cDNA, 10 *μ*M primer for each and 10 *μ*L premix (Bio-Rad Hercules, CA, USA), were analyzed via a real-time quantitative PCR system (IQ5, Biorad, CA, USA). The quantitative PCR reaction conditions were as following: 95°C for 2 min, followed by 40 cycles of 90°C for 10 s and 60°C for 40 s. The positive and negative controls were utilized to keep the accuracy of the present quantitative PCR. 2^−△△Ct^ method was used to calculate the relative mRNA levels of target genes, with *β*-actin as the reference gene. And the sequences of primers used for real-time quantitative PCR in the present study were presented in Table 1.

### 2.7. Western Blot

After being treated as described in method 2.6 and washed using PBS three times, cells derived from each group were collected and lysed using RIPA buffer (Beyotime Institute of Biotechnology, Shanghai, China) containing a protease inhibitor of 1 mM PMSF according to the supplier's instructions. Protein concentrations were determined using BCA method. Equal amounts of proteins were loaded and isolated using a sodium dodecyl sulfate-polyacrylamide gel electrophoresis (SDS-PAGE) and subsequently transferred onto a PVDF membrane. After being blocked with 5% skim milk, membranes were incubated with primary antibody (Nfr2, 1:2000 dilution; HO-1, 1:10000 dilution; NOO1, 1:20000 dilution; Abcam) and goat anti-rabbit secondary antibody were conjugated to horseradish peroxidase (HRP) (1:200 dilution) (Beyotime Institute of Biotechnology, Shanghai, China). *β*-actin was used as internal reference.

### 2.8. Immunofluorescent Assay

After being treated as described in method 2.6, cells were washed using 0.5% PBST and fixed with paraformaldehyde of 4% for 10 min, followed by being washed using 0.5% PBST three times and blocked using immunol staining blocking buffer (P0102, Beyotime Institute of Biotechnology, Shanghai, China) for 1.2 h. Then the cells were incubated with Nrf2 rabbit anti-human primary antibody (1:100 dilution) at 4°C overnight, followed by being kept at room temperature for 1 h and washed using 0.5% PBST three times. The cells were subsequently incubated with FITC labeled goat anti-rabbit secondary antibody (1:200 dilution) at 37°C in a humidified incubator for 1.5 h, followed by being washed using 0.5% PBST three times. Finally, cells were stained with Hoechst 33258 of 20 *μ*M for 5 min, followed by being washed using 0.5% PBST three times and adding antifade polyvinylpyrrolidone mounting medium (P0123, Beyotime Institute of Biotechnology, Shanghai, China). Then the slices were observed via a fluorescence microscope (Leica DMI400B).

### 2.9. Statistical Analyses

All experiments in the present work were replicated at least three times. All data were presented as means ± SD and analyzed by one-way ANOVA and* t*-test. All the statistical analyses were performed using SPSS software (version 19.0; IBM, Chicago, IL, USA). And it was considered as statistically significant in the case of *P* value smaller than 0.05.

## 3. Results

### 3.1. Effects of H_*2*_O_*2*_ Treatment on the Viability of L02 Cells

**Figure 1:**
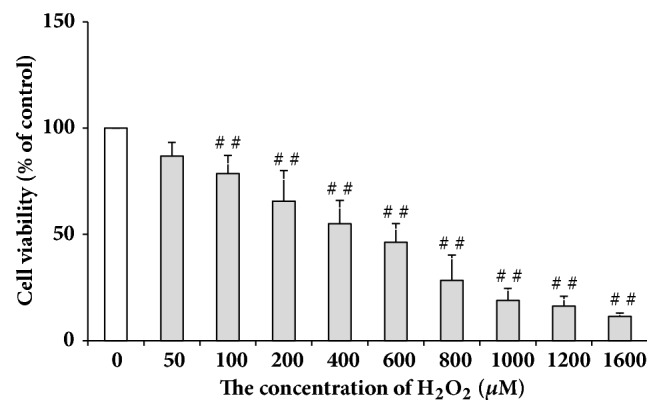
Effects of H_2_O_2_ treatment on the viability of L02 cells. Data were presented as means ± SD, n=3. ##:* P* < 0.01 (compared with control).

L02 cells were treated with H_2_O_2_ at different concentrations (100, 200, 400, 600, 800, 1000, 1200, and 1600 *μ*M, respectively) and the appropriate concentration used to induce oxidative damage was evaluated by MTT assay. As is shown in Figure 1, the cell viability decreased significantly when treatment concentration of H_2_O_2_ was equal to or more than 100 *μ*M (compared with control which is set as 100%,* P* < 0.01). In the presence of 200 *μ*M H_2_O_2_, the cell viability demonstrated a moderate decrease. Therefore, treating the L02 cells with H_2_O_2_ at a final concentration of 200 *μ*M was selected for subsequent experiments.

### 3.2. Effects of Fucoxanthin on the Viability of H_*2*_O_*2*_-Treated L02 Cells

**Figure 2:**
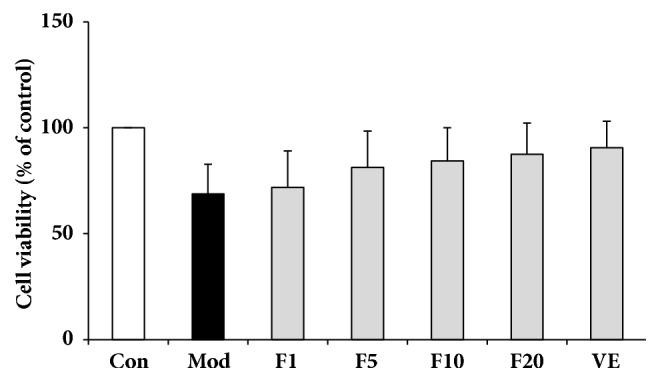
Effects of fucoxanthin on the viability of H_2_O_2_-treated L02 cells. Con: control; Mod: H_2_O_2_ model group; F1: 1 *μ*M fucoxanthin + H_2_O_2_; F5: 5 *μ*M fucoxanthin+ H_2_O_2_; F10: 10 *μ*M fucoxanthin +H_2_O_2_; F20: 20 *μ*M fucoxanthin +H_2_O_2_; VE: 50 *μ*M vitamin E + H_2_O_2_. Data were presented as means ± SD, n=3.

Effects of fucoxanthin on the viability of H_2_O_2_-treated L02 cells were also evaluated by MTT. The results were shown in Figure 2 where it could be learned that the viability increased after being treated by 50 *μ*M vitamin E (VE) or fucoxanthin (1, 5, 10, and 20 *μ*M, respectively), while there was no significant difference among the groups (*P* > 0.05).

### 3.3. Effects of Fucoxanthin on H_*2*_O_*2*_-Induced L02 Cellular LDH Leakage

**Figure 3:**
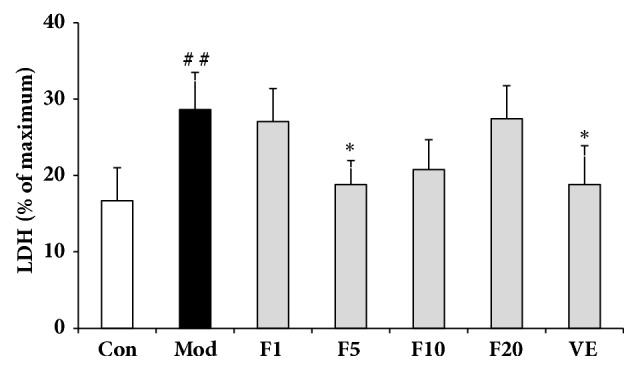
Effects of fucoxanthin on H_2_O_2_-induced L02 cellular LDH leakage. Con: control; Mod: H_2_O_2_ model group; F1: 1 *μ*M fucoxanthin + H_2_O_2_; F5: 5 *μ*M fucoxanthin+ H_2_O_2_; F10: 10 *μ*M fucoxanthin +H_2_O_2_; F20: 20 *μ*M fucoxanthin +H_2_O_2_; VE: 50 *μ*M vitamin E + H_2_O_2_. Data was shown as percentage of maximum LDH release group and presented as means ± SD, n=3. ##:* P* < 0.01, compared with control; *∗*:* P* < 0.05, compared with model group.

The leakage level of maximum LDH leakage group was set as 100% and results were presented in Figure 3. The LDH leakage rate of control group was 16.71 ± 4.48%, with model group as 28.45 ± 5.13% (compared with control,* P* < 0.01). Compared with model group, VE-treated group exhibited a leakage rate of 18.70 ± 4.98% (*P* < 0.05), with F5 group demonstrating the lowest LDH leakage (*P* < 0.05).

### 3.4. Effects of Fucoxanthin on Intracellular GSH Content in H_*2*_O_*2*_-Treated L02 Cells

**Figure 4:**
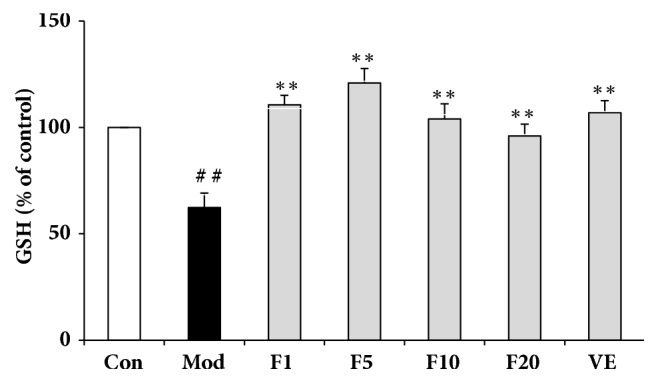
Effects of fucoxanthin on intracellular GSH content in H_2_O_2_-treated L02 cells. Con: control; Mod: H_2_O_2_ model group; F1: 1 *μ*M fucoxanthin + H_2_O_2_; F5: 5 *μ*M fucoxanthin+ H_2_O_2_; F10: 10 *μ*M fucoxanthin +H_2_O_2_; F20: 20 *μ*M fucoxanthin +H_2_O_2_; VE: 50 *μ*M vitamin E + H_2_O_2_. Data was shown as a percentage of the maximum LDH release group and presented as means ± SD, n=3. ##:* P* < 0.01, compared with control; *∗∗*:* P* < 0.01, compared with model group.

As shown in Figure 4, the GSH content in control group was set as 100% and that of model group dropped to 62.29 ± 6.92% (compared with control, *P* < 0.01). Treatment of VE (50 *μ*M) resulted in the GSH content of 106.94 ± 5.70% (compared with model group,* P* < 0.01). Pretreatment with aforementioned various concentrations of fucoxanthin resulted in the GSH content of 110.69 ± 4.39%, 120.98 ± 6.72%, 103.97 ± 7.12%, and 96.05 ± 5.59%, respectively (compared with model group,* P* < 0.01).

### 3.5. Effects of Fucoxanthin on Intracellular ROS Contents in H_*2*_O_*2*_-Treated L02 Cells

**Figure 5:**
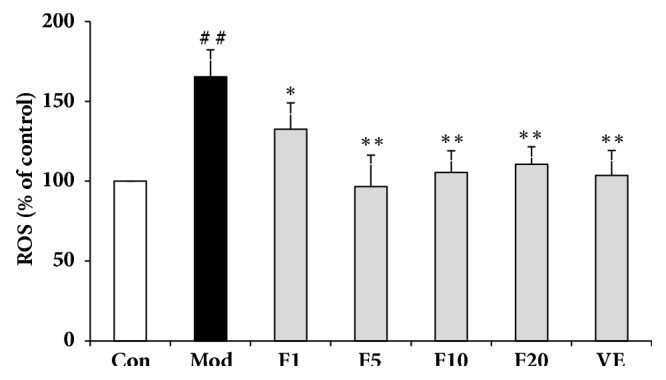
Effects of fucoxanthin on intracellular ROS contents in H_2_O_2_-treated L02 cells. Con: control; Mod: H_2_O_2_ model group; F1: 1 *μ*M fucoxanthin + H_2_O_2_; F5: 5 *μ*M fucoxanthin+ H_2_O_2_; F10: 10 *μ*M fucoxanthin +H_2_O_2_; F20: 20 *μ*M fucoxanthin +H_2_O_2_; VE: 50 *μ*M vitamin E + H_2_O_2_. The data was shown as means ± SD, n=3. ##:* P* < 0.01, compared with control; *∗*:* P* < 0.05, *∗∗*:* P* < 0.01, compared with model group.

**Figure 6:**
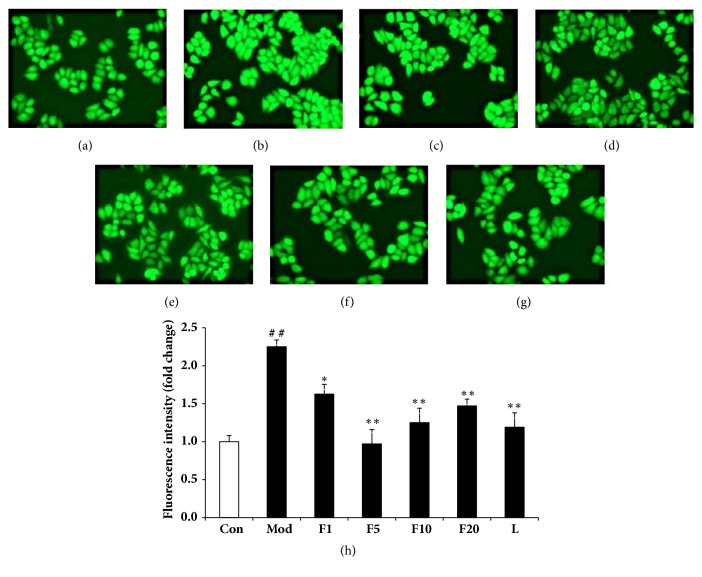
Effects of fucoxanthin on intracellular ROS contents in H_2_O_2_-treated L02 cells (×200 magnification). (a): control; (b): H_2_O_2_ model group; (c): 1 *μ*M fucoxanthin + H_2_O_2_; (d): 5 *μ*M fucoxanthin+ H_2_O_2_; (e): 10 *μ*M fucoxanthin +H_2_O_2_; (f): 20 *μ*M fucoxanthin +H_2_O_2_; (g): 50 *μ*M vitamin E + H_2_O_2_; (h): Analysis of intracellular ROS based on fluorescence microscope. ##:* P* < 0.01, compared with control; *∗*:* P* < 0.05, *∗∗*:* P* < 0.01, compared with model group.

The intracellular ROS contents were presented as a percentage of control. As shown in Figure 5, ROS content of model group increased to 165.38 ± 16.9%, compared with control (*P* < 0.01). Treatment of VE (50 *μ*M) resulted in ROS content of 103.66% ± 15.60% (compared with model group,* P* < 0.01). After being pretreated with various aforementioned concentrations of fucoxanthin, the ROS content dropped to 132.60 ± 16.55%, 96.61 ± 19.72%, 105.48 ± 13.65%, and 110.65 ± 11.00%, respectively (in comparison with model group,* P* < 0.01). The reduced fluorescence intensities, as is shown in Figure 6(h), also suggested the intracellular ROS scavenge capacity of fucoxanthin in H_2_O_2_-treated L02 cells.

### 3.6. Effects of Fucoxanthin on Nuclear Translocation of Nrf2 in H_*2*_O_*2*_-Treated L02 Cells

**Figure 7:**
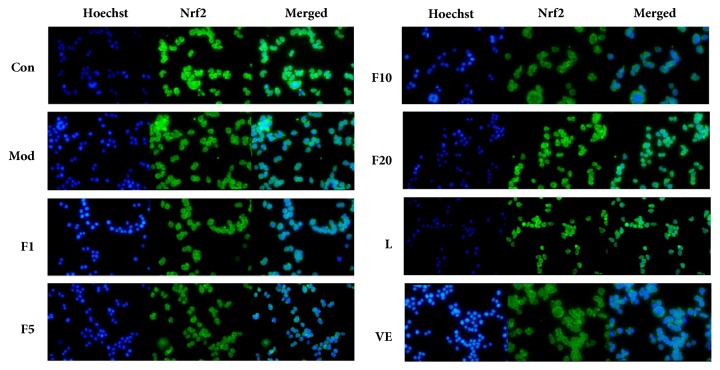
Effects of fucoxanthin on nuclear translocation of Nrf2 in H_2_O_2_-treated L02 cells (×200 magnification). Con: control; Mod: H_2_O_2_ model group; F1: 1 *μ*M fucoxanthin + H_2_O_2_; F5: 5 *μ*M fucoxanthin+ H_2_O_2_; F10: 10 *μ*M fucoxanthin +H_2_O_2_; F20: 20 *μ*M fucoxanthin +H_2_O_2_; L: H_2_O_2_ + 5 *μ*M fucoxanthin + LY294002; VE: 50 *μ*M vitamin E + H_2_O_2_; Nucleus stained with Hoechst appears as blue fluorescence in the first column and stained Nrf2 appears as green fluorescence in the second column, with the merged as the synthesis of them both.

Effects of fucoxanthin on nuclear translocation of Nrf2 in H_2_O_2_-treated L02 cells were shown in Figure 7. The expression of Nrf2 was shown in green fluorescence and blue fluorescence exhibited the nucleus stained by Hoechst 33258. Cyan represents the occurrence of nuclear transposition. It could be learned that fucoxanthin could enhance the expression of Nrf2 and promote the occurrence of Nrf2 nuclear translocation. LY294002 could inhibit the occurrence of Nrf2 nuclear translocation and reduce the expression of Nrf2.

### 3.7. Effects of Fucoxanthin on mRNA Relative Levels of Nrf2 Signaling Pathway-Related Proteins

**Figure 8:**
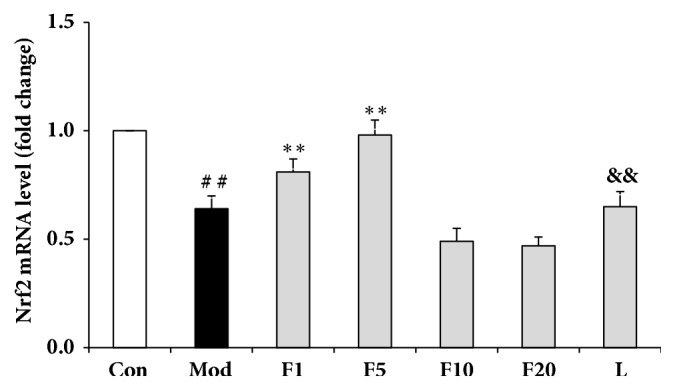
Effects of fucoxanthin on mRNA relative level of Nrf2 in H_2_O_2_-treated L02 cells. Con: control; Mod: H_2_O_2_ model group; F1: 1 *μ*M fucoxanthin + H_2_O_2_; F5: 5 *μ*M fucoxanthin+ H_2_O_2_; F10: 10 *μ*M fucoxanthin +H_2_O_2_; F20: 20 *μ*M fucoxanthin +H_2_O_2_; L: 5 *μ*M fucoxanthin + H_2_O_2_ + LY294002; Data was presented as means ± SD, n=3. ##:* P* < 0.01, compared with control; *∗∗*:* P* < 0.01, compared with model group; &&:* P* < 0.01, compared with group F5.

**Figure 9:**
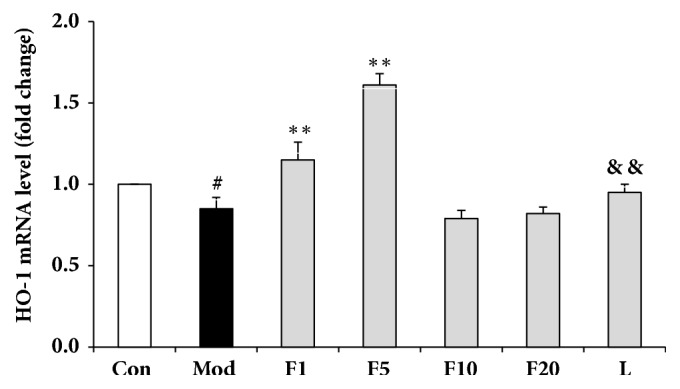
Effects of fucoxanthin on mRNA relative level of HO-1 in H_2_O_2_-treated L02 cells. Con: control; Mod: H_2_O_2_ model group; F1: 1 *μ*M fucoxanthin + H_2_O_2_; F5: 5 *μ*M fucoxanthin+ H_2_O_2_; F10: 10 *μ*M fucoxanthin +H_2_O_2_; F20: 20 *μ*M fucoxanthin +H_2_O_2_; L: 5 *μ*M fucoxanthin + H_2_O_2_ + LY294002; Data was presented as means ± SD, n=3. #:* P* < 0.05, compared with control; *∗∗*:* P* < 0.01, compared with model group; &&:* P* < 0.01, compared with group F5.

**Figure 10:**
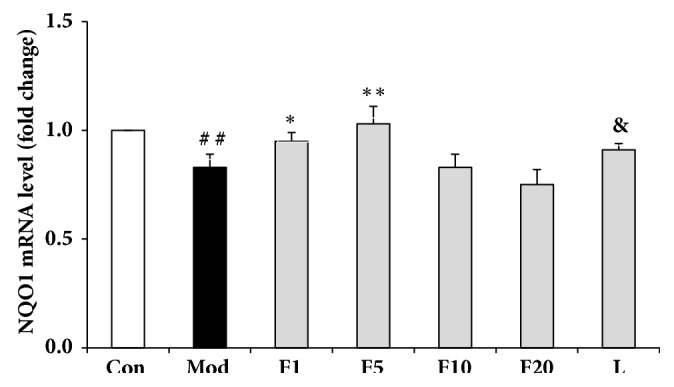
Effects of fucoxanthin on mRNA relative level of NQO1 in H_2_O_2_-treated L02 cells. Con: control; Mod: H_2_O_2_ model group; F1: 1 *μ*M fucoxanthin + H_2_O_2_; F5: 5 *μ*M fucoxanthin+ H_2_O_2_; F10: 10 *μ*M fucoxanthin +H_2_O_2_; F20: 20 *μ*M fucoxanthin +H_2_O_2_; L: 5 *μ*M fucoxanthin + H_2_O_2_ + LY294002; Data was presented as means ± SD, n=3. ##:* P* < 0.01, compared with control; *∗*:* P* < 0.05, compared with model group; *∗∗*:* P* < 0.01, compared with model group; &:* P* < 0.05, compared with group F5.

Compared with control group, mRNA relative levels of Nrf2, HO-1 and NQO1 from model group dropped significantly to 0.64 ± 0.06 (*P* < 0.01), 0.85 ± 0.07 (*P* < 0.05), and 0.83 ± 0.06 (*P* < 0.01), respectively (Figures 8–10). After being pretreated with fucoxanthin of 1 or 5 *μ*M, mRNA relative levels of Nrf2, HO-1, and NQO1 were enhanced to 0.81 ± 0.06 and 0.98 ± 0.07 (*P* < 0.01), 1.15 ± 0.11 and 1.61 ± 0.07 (*P* < 0.01), along with 0.95 ± 0.04 (*P* < 0.05), and 1.03 ± 0.08 (*P* < 0.01), respectively, in comparison with model group.

After being treated by LY294002, the mRNA relative levels of Nrf2, HO-1 and NQO1 decreased to 0.65 ± 0.07 (*P* < 0.01), 0.95 ± 0.05 (*P* < 0.01), and 0.91 ± 0.03 (*P* < 0.05), respectively, compared with group F5 (Figures 8–10).

### 3.8. Effects of Fucoxanthin on Expressions of Nrf2 Signaling Pathway-Related Proteins

**Figure 11:**
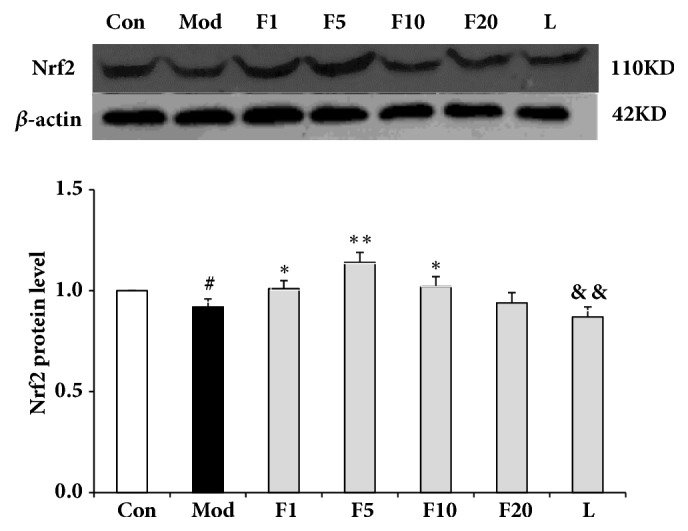
Effects of fucoxanthin on expression of Nrf2 in H_2_O_2_-treated L02 cells. Con: control; Mod: H_2_O_2_ model group; F1: 1 *μ*M fucoxanthin + H_2_O_2_; F5: 5 *μ*M fucoxanthin+ H_2_O_2_; F10: 10 *μ*M fucoxanthin +H_2_O_2_; F20: 20 *μ*M fucoxanthin +H_2_O_2_; L: 5 *μ*M fucoxanthin + H_2_O_2_ + LY294002; Data was presented as means ± SD, n=3. #:* P* < 0.05, compared with control; *∗*:* P* < 0.05, compared with model group; *∗∗*:* P* < 0.01, compared with model group; &&:* P* < 0.01, compared with group F5.

**Figure 12:**
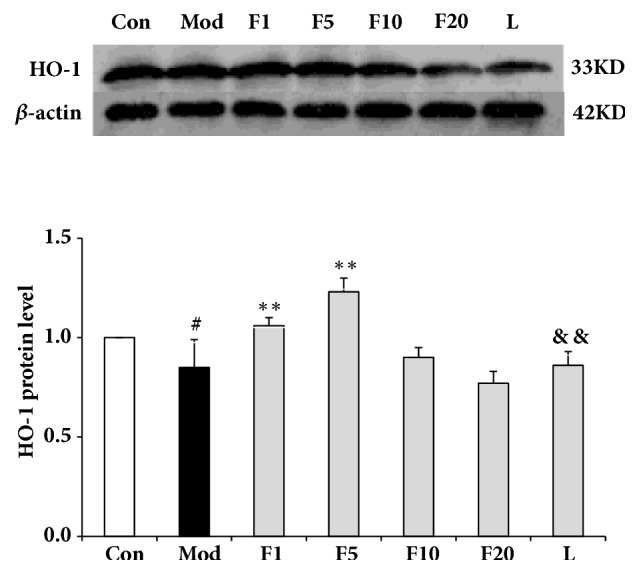
Effects of fucoxanthin on expression of HO-1 in H_2_O_2_-treated L02 cells. Con: control; Mod: H_2_O_2_ model group; F1: 1 *μ*M fucoxanthin + H_2_O_2_; F5: 5 *μ*M fucoxanthin+ H_2_O_2_; F10: 10 *μ*M fucoxanthin +H_2_O_2_; F20: 20 *μ*M fucoxanthin +H_2_O_2_; L: 5 *μ*M fucoxanthin + H_2_O_2_ + LY294002; Data was presented as means ± SD, n=3. #:* P* < 0.05, compared with control; *∗∗*:* P* < 0.01, compared with model group; &&:* P* < 0.01, compared with group F5.

**Figure 13:**
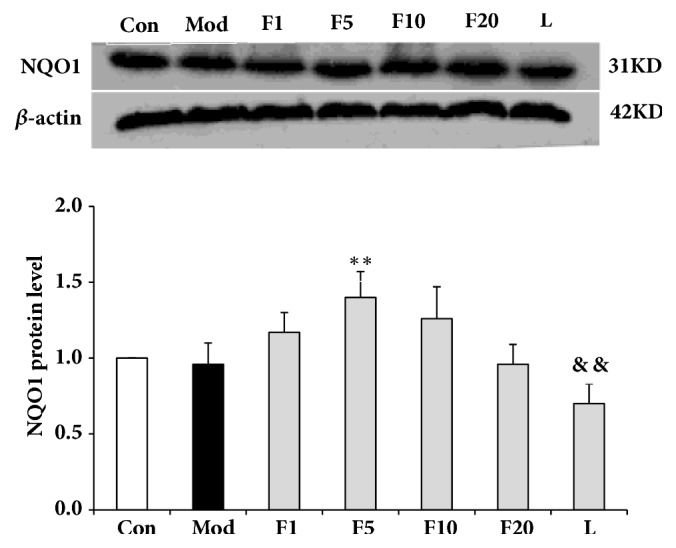
Effects of fucoxanthin on expression of NQO1 in H_2_O_2_-treated L02 cells. Con: control; Mod: H_2_O_2_ model group; F1: 1 *μ*M fucoxanthin + H_2_O_2_; F5: 5 *μ*M fucoxanthin+ H_2_O_2_; F10: 10 *μ*M fucoxanthin +H_2_O_2_; F20: 20 *μ*M fucoxanthin +H_2_O_2_; L: 5 *μ*M fucoxanthin + H_2_O_2_ + LY294002; Data was presented as means ± SD, n=3. *∗∗*:* P* < 0.01, compared with model group; &&:* P* < 0.01, compared with group F5.

As is shown in Figure 11, the expression of Nrf2 in model group dropped significantly when compared with control (*P* < 0.05). After being treated with various aforementioned concentrations of fucoxanthin, the relative protein levels of Nrf2 were enhanced to 1.01 + 0.04 (*P* < 0.05), 1.14 + 0.05 (*P* < 0.01), and 1.02 + 0.05 (*P* < 0.05), respectively, compared with model group. The relative protein level of HO-1, dropping significantly in model group when compared with control (*P* < 0.05), was presented in Figure 12. After being treated with fucoxanthin of 1 or 5 *μ*M, the relative protein level of HO-1 was enhanced to 1.06 ± 0.04 (*P* < 0.01) and 1.23 ± 0.07 (*P* < 0.01), respectively, compared with model group. As to the relative protein level of NQO1, significant difference was only found between group F5 and model group (*P* < 0.01) (Figure 13).

After being treated by LY294002, the relative protein levels of Nrf2, HO-1 and NQO1 decreased to 0.87 ± 0.05 (*P* < 0.01), 0.86 ± 0.07 (*P* < 0.01) and 0.70 ± 0.13 (*P* < 0.01), respectively, compared with group F5.

## 4. Discussion

Although H_2_O_2_ physiologically exists in living cells where it can act as a cellular signal transducer below the concentration of 1 *μ*M, at higher concentrations it might result in adverse effects such as growth arrest or cell death caused by the derived oxidative stress [28]. Cell viability can be evaluated by MTT assay and in living cells MTT could be transformed by mitochondria into formazan whose amount is positively related with living cell numbers [29]. Based on cell viability, H_2_O_2_ at a final concentration of 200 *μ*M was selected for subsequent treatment on L02 cells in the present work. The results showed that fucoxanthin pretreatment for 2 h prior to treatment of H_2_O_2_ could increase the cell viability compared with model group, suggesting the protecting effects of fucoxanthin against the cellular damages induced by H_2_O_2_. Apoptosis of hepatic cells often occur in the case of liver damage [30]; thus the pretreatment of fucoxanthin might suppress the L02 cell death induced by H_2_O_2_, though there was no significant difference between the experimental groups and model group (Figure 2) in cell viability.

LDH, a stable enzyme in the cytoplasm of all cells, could be released promptly into extracellular environment once the cell membrane is damaged [31]. Thus it may act as an important indicator of cell membrane damage extent. Pretreatment of 5 *μ*M fucoxanthin could significantly reduce the H_2_O_2_-induced intracellular LDH leakage, with effects comparable to VE of 50 *μ*M, suggesting a role of fucoxanthin in protecting the integrity of cell membranes and counteracting the H_2_O_2_-induced oxidative damage. Similarly, pretreatment of fucoxanthin also led to decline of intracellular ROS. The inhibition of ROS generation by fucoxanthin might be due to its two hydroxyl groups in the ring structure and the number of hydroxyl groups has been reported correlated with inhibited ROS production [32]. GSH, an abundant thiol in liver, could scavenge free radicals or serve as a substrate to glutathione peroxidase (GSHPx) and glutathione s-transferase (GST) to detoxify the H_2_O_2_-induced damages [33]. The pretreatment of fucoxanthin could significantly reverse the decline of GSH induced by H_2_O_2_, consistent with what was reported by Zheng J et al. [34]. As to the effects on GSH and intracellular ROS levels, fucoxanthin performed better than VE of 50 *μ*M did. The excellent effects of fucoxanthin aforementioned might be due to its higher antioxidant activity which is based on its allenic bond, epoxide group and hydroxyl group [35].

Lowe et al. found that *β*-carotene protected HT29 cells from H_2_O_2_-induced damaging effects only at low concentrations of about 2-3 *μ*M instead of higher concentration of 4-10 *μ*M where the protection ability was rapidly lost [36], similar to what was found in H_2_O_2_-induced DNA damage in HepG2 cells by Woods et al. [37]. In the present work, fucoxanthin at concentrations higher than 5*μ*M also presented lower effects against oxidative damage induced by H_2_O_2_ in a similar manner. *β*-Carotene may act as a pro-oxidant at higher concentrations. In vitro, whether *β*-carotene plays as a pro-oxidant or oxidant depends on oxygen tension and its concentration [38]. However, *β*-carotene exhibits pro-oxidant effects at 2.5 *μ*M in LS-174 cells, presumably due to the capability difference of cell to incorporate the carotenoid [39]. Fucoxanthin demonstrated higher DPPH radical scavenging capacity than *β*-carotene [40] but lower reducing power than ascorbic acid [41]. Superior antioxidant activity of fucoxanthin than *β*-carotene, along with slightly less potency than ascorbic acid, was also reported and ascorbic acid of 1 *μ*M significantly reduced the H_2_O_2_-induced sister chromatid exchanges [35]. Fucoxanthin of 5 *μ*M demonstrated excellent cytoprotective effects against the H_2_O_2_-induced oxidative damage in the present work. Hence, based on the aforementioned data, fucoxanthin possesses the prominent antioxidant ability to protect against the cellular damages induced by H_2_O_2_ and might be a potential therapeutic agent for treating or preventing diseases related to oxidative stress.

Since large quantities of ROS are produced continuously throughout life, antioxidant mechanisms are essential for cells to maintain redox homoeostasis [42]. There are many key regulators, such as nuclear factor- (erythroid-derived 2-) like 2 (Nrf2), to protect against oxidative damage. Nrf2 plays a vital role in maintaining cellular redox homoeostasis by activating a variety of cytoprotective enzymes including quinine oxidoreductase 1 (NQO1) and heme oxygenase-1 (HO-1) to attenuate liver injury [43]. Under stressed conditions, Nrf2 leaves from its inhibitor of CNC homology- (ECH-) associated protein 1 (Keap1) and is then translocated from the cytoplasm to the nucleus where it could transcriptionally activate its targeted cytoprotective enzymes by binding to the antioxidant response element (ARE) located in their promoter regions [44]. Thus Nrf2 acts as a molecular switch to activate the cytoprotective enzymes defending against oxidative stress. The activation of Nrf2 by some dietary phytochemicals demonstrated chemopreventive effects to suppress oxidative stress [13, 45]. HO-1 has been reported to exert its cytoprotective effects via reducing intracellular pro-oxidant levels while enhancing the levels of carbon monoxide and bilirubin [46]. And the elevated carbon monoxide and bilirubin contribute to antiapoptotic effects and fighting against cellular injury induced by free radical, respectively [47, 48]. NQO1 can be easily induced by various stresses including oxidative stress [49]. The induced high levels of it can be mediated by Nrf2 or Ah receptor [50]. In the present study, pretreatment of 5 *μ*M fucoxanthin prior to exposure to H_2_O_2_ of 200 *μ*M enhanced the mRNA and protein relative levels of Nrf2, HO-1 and NQO1, consistent with what was reported by Liu et al. [27]. And the nuclear translocation of Nrf2 was also observed (Figure 7). It could be speculated that fucoxanthin might activate the nuclear translocation of Nrf2 and then upregulate the expressions of its targeted genes of HO-1 and NQO1, thereby playing the role of suppressing the oxidative damage through the Nrf2/ARE signaling. Thus it seems that Nrf2 plays a vital role in protecting against the H_2_O_2_-induced liver injuries [51].

LY294002 could selectively inhibit the PI3K nexus by competitively and reversibly acting on the ATP-binding site of PI3K [52]. The participations of PI3K pathway in cell survival, proliferation, migration, and metabolism have been reported [53]. Since Nrf2 signaling pathway targeting the genes of HO-1 and NQO1 may be mediated by PI3K pathway [54], protein kinase C (PKC) pathway [55], c-jun N-terminal kinase (JNK) pathway [56], or ERK pathway [57], etc., LY294002 was used in group L in the present work to investigate whether the Nrf2 signaling pathway involving the antioxidant activity of fucoxanthin was mediated by PI3K pathway. The results of group L exhibited that the mRNA and protein relative levels of Nrf2, HO-1 and NQO1 were significantly downregulated, weakening the antioxidant activity of fucoxanthin. Moreover, the nuclear translocation of Nrf2 was also inhibited by LY294002. Hence, the present work suggests that fucoxanthin may exert its antioxidant effects in L02 cells against H_2_O_2_-induced oxidative damage through PI3K-dependent activation of Nrf2 signaling, while the exact mechanism of action still needs further study.

It must be noted that pretreatment by 5 *μ*M fucoxanthin in the present work demonstrated the optimal effects on the parameters such as LDH release, GSH content, and intracellular ROS content, along with the induction of Nrf2, HO-1, and NQO1. Interestingly, it does not work in a concentration-dependent manner, inconsistent with a previous study using a monkey kidney fibroblast line (Vero) [58]. Similarly, some carotenoids, such as *β*-carotene and lycopene, also presented lower antioxidant or antitumor activities in vitro at concentrations higher than 10 *μ*M [59, 60]. Cao and Cutler also demonstrated that high concentrations of antioxidants, such as Trolox and uric acid, may not conducive to defense against oxidative stress [61]. It has been reported that many antioxidants at high concentrations might turn out to serve as pro-oxidants and induce large amount of ROS which may lead to oxidative stress and cytotoxicity [62, 63]. There might be three possible explanations for such a bell-shaped effect. Firstly, as a carotenoid, fucoxanthin at higher concentrations (10 *μ*M and 20 *μ*M) in the present work might be able to cause the L02 cell membrane more permeabilized in a similar manner to *β*-carotene [36], consistent with the results of LDH leakage (Figure 3). Secondly, 10 *μ*M might exceed the capability of L02 cells to incorporate fucoxanthin, just like *β*-carotene utilized in human colon adenocarcinoma cell lines [39]. Thirdly, fucoxanthin at higher concentrations (more than 10 *μ*M) might play a role of pro-oxidant instead of antioxidant, and its pro-oxidant activity may arise from the 5,6-monoepoxide which has been shown to undergo ring opening reactions resulting from attacking nucleophiles [64]. However, the mechanism for such a bell-shaped effect needs further investigations to elucidate.

In general, fucoxanthin demonstrated excellent antioxidant activity in defending against the H_2_O_2_-induced oxidative damage in L02 cells. However, data from the present study should be interpreted with caution since there is a limitation that only the cell experiments were performed. Furthermore, it has been reported that many compounds demonstrated lower antioxidant and disease-prevention activities in vivo than they did in vitro, which is called as “the antioxidant paradox” [63]. Therefore, further intensive research is needed to elucidate the exact antioxidant mechanism of fucoxanthin. However, the prominent effects against H_2_O_2_-induced oxidative damage demonstrated by fucoxanthin suggest that it might be a promising agent in protecting against oxidative stress-related diseases in future.

## 5. Conclusions

In conclusion, the results in the present work suggest that fucoxanthin exerts its cytoprotective effects in L02 cells against H_2_O_2_-induced oxidative damage, which may be through PI3K-dependent activation of Nrf2 signaling.

## Figures and Tables

**Table 1:** Primers used for real-time quantitative PCR.

**Target**	**Primer Sequence (5'–3')**
Nrf2	F: TCCAGTCAGAAACCAGTGGAT
	R: GAATGTCTGCGCCAAAAGCTG

HO-1	F: CCAGCAACAAAGTGCAAGAT
	R: GTGTAAGGACCCATCGGAGA

NQO1	F: GAAGAGCACTGATCGTACTGGC
	R: GGATACTGAAAGTTCGCAGGG

*β*-actin	F: ATTGCCGACAGGATGCAGA
	R: GAGTACTTGCGCTCAGGAGGA

## Data Availability

The data used to support the findings of this study are included within the article.
